# High-Volume Online Hemodiafiltration: Does Patient Phenotype Matter?

**DOI:** 10.3390/jcm15207922

**Published:** 2026-10-14

**Authors:** Rosa Ramos, Francisco Maduell, Jose Jesús Broseta

**Affiliations:** 1 Department of Nephrology, Institut Clínic de Nefrologia i Urologia (ICNU), Hospital Clínic de Barcelona, C/Villarroel 170, 08036 Barcelona, Spain; fmaduell@clinic.cat (F.M.); jjbroseta@clinic.cat (J.J.B.); 2 Faculty of Medicine and Health Sciences, Universitat de Barcelona, 08036 Barcelona, Spain; 3 Institut d’Investigacions Biomèdiques August Pi i Sunyer (IDIBAPS), 08036 Barcelona, Spain

**Keywords:** hemodiafiltration, hemodialysis, convection volume, patient phenotype, mortality, precision medicine

## Abstract

Online hemodiafiltration (OL-HDF) has been associated with improved clinical outcomes compared with conventional hemodialysis in randomized trials, particularly in studies achieving high convection volumes. Whether specific patient phenotypes derive greater benefit from OL-HDF remains uncertain. This narrative review examines the available evidence regarding potential clinical phenotypes associated with enhanced benefit from high-volume OL-HDF, including older adults, anuric patients, individuals with heart failure, patients with poor intradialytic hemodynamic tolerance, frail patients, those with increased inflammatory burden, and incident dialysis patients. Current randomized evidence has not detected convincing treatment-effect modification across the clinical subgroups examined; however, most subgroup analyses were not designed or powered to establish equivalence of treatment effects. Higher achieved convection volumes have been associated with improved outcomes, although these observational or post-randomization associations should not be interpreted as proof of a causal dose–response relationship. Nevertheless, several high-risk clinical scenarios may be associated with greater absolute clinical benefit because of an elevated baseline risk of adverse outcomes. Available data suggest that personalization of OL-HDF should focus less on selecting eligible phenotypes and more on optimizing treatment delivery according to patient characteristics, treatment goals, and technical feasibility. Future research should explore patient-centred outcomes and identify strategies to maximize the benefits of high-volume OL-HDF within a precision medicine framework.

## 1. Introduction

Online hemodiafiltration (OL-HDF) has become an increasingly important kidney replacement therapy following randomized evidence demonstrating improved clinical outcomes compared with conventional high-flux HD in selected populations. However, patients receiving maintenance dialysis are markedly heterogeneous in age, comorbidity, residual kidney function, cardiovascular vulnerability, treatment tolerance, and technical capacity to achieve high convection volumes. Whether these characteristics modify the relative effect of OL-HDF, influence absolute treatment benefit, or primarily affect treatment feasibility remains uncertain.

Current evidence suggests that achieving high convection volumes is more important than identifying a particular patient subgroup. In the most recent individual-patient meta-analysis, no evidence for a differential effect across subgroups was noted, but also that a graded relationship between convection volume and mortality risk was apparent: as the volume increased, the mortality risk decreased [1]. An earlier pooled analysis said the same thing more bluntly, stating that “There was no evidence for a differential effect in subgroups,” while the biggest survival signal sat in the highest delivered convection-volume group [2].

Therefore, there is currently no clearly established clinical phenotype that derives a consistently greater benefit from OL-HDF [3]. The practical pattern is that patients who can receive and sustain higher convection volumes seem to do better. One body-size analysis found that standardizing dose by body surface area or total body water preserved the survival signal, whereas body weight or BMI did not, and another cohort found that centre practice and patient clinical conditions both drove whether patients reached target substitution volumes [4,5].

Although these findings argue against the existence of a single phenotype that derives a disproportionate benefit from OL-HDF, they do not exclude the possibility that certain clinical characteristics may influence the magnitude or nature of treatment benefits.

These observations also suggest that personalization of OL-HDF may rely less on selecting which patients should receive the therapy and more on tailoring how OL-HDF is prescribed and delivered. Convective dose, vascular access performance, treatment time, hemodynamic tolerance, and sodium and volume management may therefore represent key components of a precision-medicine approach.

Randomized trials have not been powered to identify treatment interactions across specific patient subgroups, and many clinically relevant outcomes, including cardiovascular events, hospitalization, symptom burden, hemodynamic tolerance, and quality of life, may not be fully captured by mortality analyses alone. Three distinct concepts should therefore be distinguished when interpreting phenotype-specific findings: the average treatment effect observed in the overall trial population, relative effect modification across patient subgroups, and absolute treatment benefit, which depends on both relative treatment effect and baseline risk. Failure to detect a statistically significant treatment-by-subgroup interaction should not be interpreted as evidence that treatment effects are identical across subgroups, particularly because most available trials were not designed or powered to detect such interactions. Conversely, patients at higher baseline risk may derive a larger absolute benefit even in the absence of relative effect modification. Consequently, exploring high-risk phenotypes remains clinically relevant, not to identify exclusive beneficiaries, but to better understand which patients might derive the greatest absolute benefit from high-volume OL-HDF.

This narrative review examines the evidence supporting potential clinical phenotypes relevant to OL-HDF and distinguishes three related but different questions: whether a characteristic modifies the relative treatment effect, whether high baseline risk may result in greater absolute benefit, and whether patient characteristics influence the ability to deliver effective convective therapy.

Throughout this review, the term “clinical phenotype” is used pragmatically, acknowledging that many of these categories overlap and do not represent biologically distinct populations. Rather, they reflect clinical scenarios in which treatment priorities and OL-HDF prescription may differ. The clinical characteristics, biological rationale, and available evidence supporting each proposed phenotype are summarized in Table 1.

## 2. Methods: Literature Search and Evidence Synthesis

This narrative review was informed by a structured literature search conducted in MEDLINE PubMed up to 31 July 2026. Search terms included combinations of “online hemodiafiltration”, “hemodiafiltration”, “convection volume”, “mortality”, “age”, “older adults”, “frailty”, “heart failure”, “intradialytic hypotension”, “residual kidney function”, “anuria”, “inflammation”, and “incident dialysis”. We considered randomized controlled trials, individual-participant-data meta-analyses, systematic reviews, consensus documents, and observational studies addressing OL-HDF and the clinical scenarios discussed in this review. Priority was given to randomized evidence, individual-participant-data analyses, and recent systematic reviews. Observational studies were included when randomized phenotype-specific evidence was unavailable or when they provided information on treatment feasibility or populations insufficiently represented in randomized trials.

Because the aim of this narrative review was to provide a clinically oriented interpretation of the available evidence rather than a systematic evidence synthesis, study selection was not performed according to a predefined systematic-review protocol, and no formal risk-of-bias assessment or quantitative meta-analysis was undertaken.

**Figure 1:**
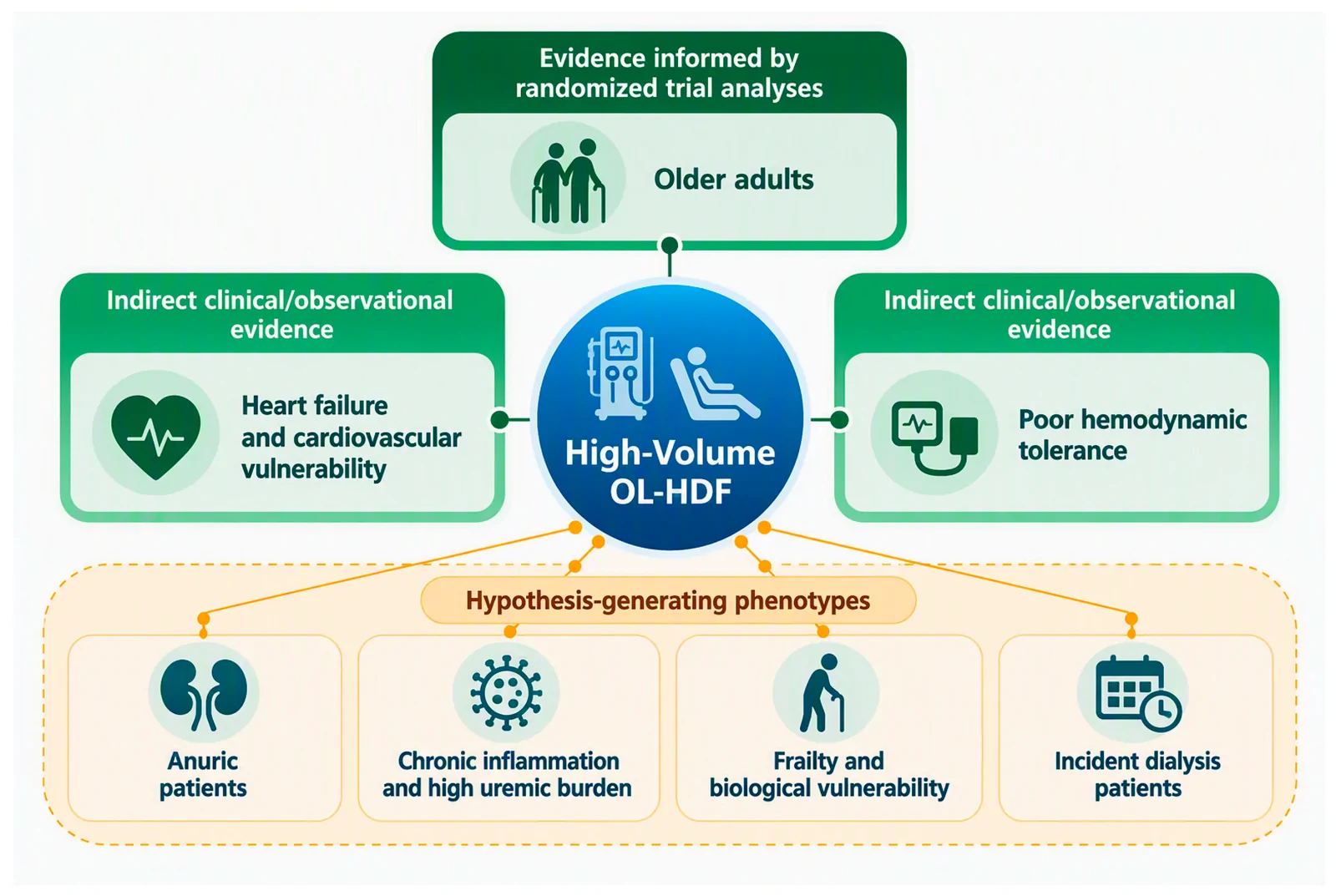
Proposed patient phenotype framework for individualized consideration of high-volume OL-HDF according to the strength of supporting evidence.

Generative artificial intelligence (Microsoft Copilot) was used exclusively to assist in the design of Figure 1. No AI tools were used for literature review, data analysis, data interpretation, or manuscript writing.

## 3. Technical Framework: What Constitutes High-Volume OL-HDF?

OL-HDF combines diffusive and convective solute transport. In post-dilution OL-HDF, sterile substitution fluid is infused downstream from the dialyzer, whereas in pre-dilution OL-HDF it is infused upstream, reducing hemoconcentration within the dialyzer but requiring larger substitution volumes to achieve comparable solute removal. Substitution volume refers to the volume of sterile replacement fluid infused during the treatment, whereas total convection volume additionally includes net ultrafiltration. These quantities should therefore not be used interchangeably. Throughout this review, terminology and reported volumes follow the definitions used in the original studies, and post-dilution OL-HDF is specified where relevant.

The concept of “high-volume” OL-HDF requires careful definition because absolute convection volume is strongly influenced by patient size and treatment characteristics. Earlier studies suggested that the relationship between convection volume and outcome may be better characterized when convection is indexed to body surface area or total body water rather than body weight or body mass index [4,6]. Accordingly, a fixed absolute convection-volume threshold may not represent an equivalent convective exposure across patients with markedly different body composition. In fact, actual recommendations based on secondary analysis and recent trials state a minimum amount of convection of 23 L adjusted to body surface area per session to maximize the benefit of the technique [1].

Importantly, achieved convection volume is not a randomized treatment exposure. It is a post-randomization measure influenced by vascular access performance, blood-flow rate, treatment duration, body size and composition, ultrafiltration requirements, hemodynamic tolerance, adherence, and centre expertise. Associations between higher achieved convection volumes and improved outcomes may therefore be affected by patient selection, treatment feasibility, and residual confounding. Consequently, observational gradients across achieved convection-volume categories should not be interpreted as establishing a causal dose–response relationship.

This distinction does not diminish the clinical importance of delivering adequate convection when OL-HDF is prescribed. Rather, it emphasizes that randomized comparisons of OL-HDF versus HD and observational analyses according to achieved convection volume address different causal questions and should be interpreted separately.

## 4. Age and OL-HDF: Why Older Patients May Matter Most

Advanced age has emerged as a clinically relevant characteristic when considering the potential benefits of OL-HDF, largely because older patients represent one of the highest-risk populations receiving dialysis. Older patients carry a greater burden of cardiovascular disease, inflammation, and dialysis-related complications, several of which have been associated with middle-molecule retention and may therefore be particularly relevant targets for enhanced convective clearance with OL-HDF. These patients may also derive clinically relevant benefits beyond survival, including improved hemodynamic stability, better symptom control, and potentially fewer cardiovascular events [7].

The ESHOL trial provides important evidence supporting the clinical value of OL-HDF in older patients [8]. Although formal interaction testing did not demonstrate age-specific treatment effects, subgroup analyses suggested that the reduction in all-cause mortality was numerically greater among older individuals, supporting the possibility of a larger absolute clinical benefit in this population. More recent evidence from CONVINCE demonstrated lower all-cause mortality with high-volume OL-HDF compared with high-flux HD. Cause-specific analyses should be interpreted more cautiously: infection-related mortality was lower when including COVID-19 cases, whereas cardiovascular mortality and the composite of fatal and nonfatal cardiovascular events were not significantly reduced [9].

Importantly, pooled analyses of randomized controlled trials have consistently shown that the relative mortality benefit of OL-HDF is preserved across age groups, with no convincing evidence of a treatment-by-age interaction when adequate convection volumes are achieved [1,2]. This suggests that age itself should not be used to restrict access to OL-HDF. Interestingly, individualized treatment-effect analyses have suggested that younger patients may accumulate a greater number of life-months gained with OL-HDF [10]. However, this observation most likely reflects their longer remaining life expectancy rather than a superior biological response to the therapy itself. Consequently, advanced age should perhaps be regarded less as a predictive phenotype than as a clinical scenario in which maximizing treatment tolerance and delivering effective OL-HDF become particularly relevant.

Thus, while younger patients may experience larger gains in survival time, older patients may derive equally—or even more—meaningful absolute clinical benefits because of their substantially higher baseline risk.

## 5. Anuric Patients

Residual kidney function is one of the strongest predictors of survival in dialysis populations. Therefore, the disappearance of native kidney clearance may increase reliance on extracorporeal removal of middle molecules and protein-bound toxins, theoretically amplifying the value of convective therapies.

Patients with absent residual kidney function may represent another biologically plausible phenotype for OL-HDF. In anuric patients, extracorporeal therapy becomes the principal mechanism for removal of dialyzable uraemic solutes. OL-HDF enhances the clearance of selected middle-molecular-weight solutes compared with conventional HD, whereas removal of larger inflammatory mediators and protein-bound uraemic toxins is more limited and depends on molecular characteristics and membrane properties.

Supporting this hypothesis, a randomized study demonstrated that the anti-inflammatory effects of OL-HDF were more pronounced in anuric patients than in those with preserved residual kidney function, suggesting that the biological impact of convective therapies may increase as native kidney clearance disappears [11]. Furthermore, anuria is associated with greater inflammation, higher cardiovascular risk, poorer phosphate control, and increased mortality, characteristics that overlap with several mechanisms potentially targeted by OL-HDF. While current evidence remains insufficient to establish anuria as a definitive predictive phenotype, patients lacking residual kidney function constitute a clinically attractive population for future phenotype-oriented studies.

## 6. Patients with Heart Failure and Cardiovascular Vulnerability

Patients with heart failure and high cardiovascular burden represent another clinically attractive phenotype for OL-HDF. Cardiovascular disease remains the leading cause of death in dialysis populations. Furthermore, patients with heart failure are particularly vulnerable to volume overload, recurrent intradialytic hemodynamic instability, and chronic inflammation, pathophysiological mechanisms that may be influenced by convective dialysis therapies [12]. These mechanisms may be amenable to the enhanced convective clearance and improved hemodynamic stability associated with OL-HDF.

Although randomized trials have not demonstrated a specific treatment interaction according to heart failure status, the reduction in cardiovascular mortality observed in studies such as ESHOL and CONVINCE provides biological plausibility for a greater absolute benefit in patients with established cardiovascular disease [8,9]. Nevertheless, dedicated studies focusing specifically on patients with heart failure are lacking, and this phenotype should currently be regarded as hypothesis-generating rather than evidence-based.

Moreover, in these patients, personalization probably extends beyond convective therapy itself and includes individualized sodium prescription, fluid removal strategies, and optimization of intradialytic hemodynamic tolerance.

## 7. Poor Hemodynamic Tolerance

Patients with poor intradialytic hemodynamic tolerance may represent a clinically relevant subgroup for OL-HDF. The physiological advantages of OL-HDF, including improved thermal balance and greater hemodynamic stability during dialysis sessions, provide a biologically plausible rationale for this benefit. In the FRENCHIE randomized trial involving patients over 65, overall adverse events were similar between treatment modalities, but session-level analysis showed “fewer episodes of intradialytic symptomatic hypotension and muscle cramps” with OL-HDF; however, health-related quality of life, morbidity, and mortality were not different [13]. This session-level reduction in intradialytic events, however, should not be interpreted as evidence of improvement in patient-level morbidity or survival.

Although evidence remains limited, these findings suggest that OL-HDF may improve intradialytic tolerance in patients prone to hypotension and treatment-related symptoms, an area that deserves further investigation in dedicated clinical studies. Nevertheless, these benefits should probably be interpreted within a broader strategy that also includes dialysate temperature, ultrafiltration profiling, and individualized fluid management [14].

## 8. Frailty and Biological Vulnerability

Patients with frailty and increased biological vulnerability may represent another clinically relevant phenotype for OL-HDF. Frailty is a multidimensional condition characterized by reduced physiological reserve, sarcopenia, inflammation, and increased susceptibility to physiological stressors [15,16]. Importantly, frailty reflects biological vulnerability more accurately than chronological age alone and is independently associated with adverse outcomes such as mortality, cardiovascular events, hospitalization, and impaired quality of life in dialysis populations [15,17].

Beyond survival, frailty is strongly associated with patient-centred outcomes, including prolonged post-dialysis recovery time, reduced physical performance, and diminished resilience to intradialytic stress in HD [17]. Dialysis strategies that improve hemodynamic stability and attenuate inflammatory activation, as reported with high-volume OL-HDF, may therefore be particularly advantageous in frail patients by reducing cardiovascular stress, improving symptom burden, and potentially preserving functional status.

For frail older patients, there is another signal: in very older patients over 75, OL-HDF blunted the decline in nutritional markers seen with HD and raised HDL cholesterol [18]. In fact, frailty identifies individuals with very high baseline risk. In such populations, even modest relative reductions in mortality or cardiovascular events—such as those observed with high-volume OL-HDF in randomized trials—may translate into clinically meaningful absolute benefits. These benefits may include fewer hospitalizations, reduction in cardiovascular complications, and improvements in quality of life, outcomes that are highly relevant to frail dialysis patients and may not be fully captured by mortality-based analyses alone.

Together, these data suggest that high-volume OL-HDF may provide meaningful clinical benefits even in frail dialysis patients, addressing key pathophysiological mechanisms associated with biological vulnerability.

Even more, patient-reported outcomes are particularly relevant in this context. In the published CONVINCE patient-reported outcome analysis, a moderate positive effect on patients’ quality of life, most pronounced with respect to their cognitive function was shown [19]. These findings emphasize that treatment evaluation should extend beyond mortality and cardiovascular events to include symptom burden, physical function, recovery, and health-related quality of life.

## 9. Patients with Inflammation and High Uremic Burden

Patients characterized by systemic inflammation and a high burden of retained middle molecules may represent another biologically plausible target population for OL-HDF. Persistent inflammation contributes to erythropoietin resistance, protein-energy wasting, cardiovascular disease, and poor clinical outcomes in dialysis patients. Given its enhanced removal of middle-molecular-weight solutes, OL-HDF may offer particular advantages in this setting.

A randomized trial found the anti-inflammatory effect was more pronounced in anuric patients [11]. A 24-RCT meta-analysis found lower CRP, phosphorus, β2-microglobulin, and weekly erythropoietin requirements with OL-HDF, and the bigger the convection volume, the larger the CRP and β2-microglobulin drop [20]. A smaller randomized crossover study also found better phosphate, PTH, CRP, and HDL profiles, plus lower erythropoietin and phosphate-binder doses [21].

For anemia, multiple studies point the same way: the 2011 crossover trial [21] and the 2026 meta-analysis [20] found that OL-HDF reduced weekly erythropoietin requirements, and reported that erythropoietin and phosphate-binder doses could be reduced. The 2018 anemia-focused study also found better hemoglobin, hematocrit, iron status, and lower CRP in OL-HDF patients than in standard HD [22].

Phosphate control looks quite favourable too. The 2011 randomized crossover study [21] and the 2026 meta-analysis [20] found lower phosphate and pooled intact PTH alongside higher HDL and lower triglycerides with OL-HDF.

Although these findings support a biologically plausible advantage of OL-HDF in patients with high uremic burden, evidence demonstrating superior effects on hard clinical outcomes within these specific subgroups remains limited. In fact, although improvements in inflammatory and biochemical markers are encouraging, the translation of these surrogate/biochemical endpoints into clinically meaningful benefits for individual patients remains uncertain.

## 10. Incident Dialysis Patients: An Opportunity for Early Implementation

Rather than representing a distinct biological phenotype, incident patients may constitute a strategic opportunity for early implementation of high-volume OL-HDF before years of dialysis-related cardiovascular accumulated stress. Unlike prevalent patients, who may already have accumulated years of exposure to conventional hemodialysis-related cardiovascular stress, inflammation, and middle-molecule retention, incident patients offer an opportunity to initiate a more convective dialysis strategy from the outset. Although randomized trials have not specifically addressed this question, observational studies have consistently suggested promising outcomes associated with OL-HDF in incident populations.

Several cohort studies have reported improved survival among incident patients treated with high-volume OL-HDF compared with conventional high-flux hemodialysis. In a multicenter cohort study, Imamović and colleagues observed better survival in patients receiving higher-volume OL-HDF than in those treated with low-volume OL-HDF or high-flux HD [23]. Similarly, analyses from Romanian and European cohorts demonstrated survival advantages associated with OL-HDF, despite the inherent limitations of non-randomized designs [24,25]. These findings were further supported by a propensity-matched analysis reporting improved survival among incident patients treated with high-volume OL-HDF and by a subsequent multicenter study showing reductions in all-cause and cardiovascular mortality in incident hemodialysis patients receiving OL-HDF [26].

Evidence from a large international cohort including 85,117 patients showed that the survival benefit associated with OL-HDF was already evident among incident dialysis patients, with a 22% lower mortality risk compared with conventional hemodialysis (HR 0.78, 95% CI 0.74–0.82), supporting the early implementation of high-volume OL-HDF from dialysis initiation [27]. This observation was further reinforced by a propensity-matched study including more than 18,000 incident patients, which reported 20% and 29% reductions in all-cause and cardiovascular mortality, respectively, among patients treated with OL-HDF [28].

Taken together, these data suggest that the potential benefits of OL-HDF are not restricted to prevalent survivors and may extend to patients initiating kidney replacement therapy. While the available evidence does not establish incident status as a predictive phenotype for treatment response, it raises the possibility that earlier exposure to high-volume OL-HDF could maximize cumulative clinical benefit over time. Future randomized studies are needed to determine whether a strategy of OL-HDF-first dialysis should be considered from dialysis initiation.

The candidate phenotypes discussed throughout this review and their potential relationship with high-volume OL-HDF are schematically summarized in Figure 1.

## 11. From Phenotypes to Precision OL-HDF: Limitations and Future Directions

The consistency of subgroup findings across the major randomized studies and individual-patient meta-analyses is summarized in Table 2.

Several limitations should be considered when interpreting the available evidence on phenotype-specific responses to OL-HDF. First, most randomized trials were designed to estimate an average treatment effect rather than to assess treatment-effect heterogeneity, and subgroup analyses were frequently exploratory, post hoc, or insufficiently powered to detect treatment-by-subgroup interactions. Therefore, the absence of a statistically significant interaction should not be interpreted as evidence of an identical relative treatment effect across phenotypes. Second, evidence supporting several of the clinical scenarios discussed in this review, including frailty, heart failure, anuria, inflammatory burden, and incident dialysis is largely indirect and relies on observational analyses, surrogate endpoints, or biological plausibility rather than randomized comparisons specifically designed to test phenotype-specific benefit. Third, achieved convection volume is a post-randomization measure influenced by patient characteristics, body size and composition, vascular access, blood flow, treatment duration, hemodynamic tolerance, adherence, and centre expertise. Associations between higher achieved convection volumes and improved outcomes may therefore be affected by selection and residual confounding and should not be interpreted as establishing a causal dose–response relationship. Finally, definitions of the proposed phenotypes and the outcomes assessed vary substantially across studies, further limiting direct comparisons and the formulation of phenotype-specific treatment recommendations.

Within these limitations, the available evidence suggests that a simple phenotype-based approach is unlikely to capture the full heterogeneity of treatment response and feasibility in OL-HDF. This provides the rationale for moving from conventional clinical phenotypes toward a broader precision-dialysis framework. However, the absence of a clearly identifiable OL-HDF-responsive phenotype should not be interpreted as evidence that all patients derive identical benefits from convective therapies. Rather, it may reflect the limitations of categorizing dialysis patients according to broad clinical characteristics such as age, heart failure, frailty, or residual kidney function. While these categories are clinically meaningful and useful for risk stratification, they may be insufficiently granular to capture the complex biological and clinical heterogeneity that characterizes contemporary dialysis populations.

The failure to identify treatment-by-subgroup interactions does not necessarily imply that personalization is irrelevant. Indeed, emerging precision-medicine frameworks suggest that biological and clinical heterogeneity may be insufficiently captured by traditional phenotype classifications [29,30,31]. The candidate phenotypes reviewed in this article share a common feature: they identify patients at elevated risk of adverse outcomes and therefore potentially greater absolute benefit from optimized dialysis strategies. However, none has consistently demonstrated a differential relative response to OL-HDF in randomized studies. This distinction may be important, as the variables currently used to define phenotypes may not adequately represent the mechanisms driving response to therapy.

The concept of precision medicine offers a broader framework for understanding this challenge. Instead of asking whether a particular category of patients should receive OL-HDF, future research may need to focus on determining how OL-HDF can be optimally prescribed and delivered for a given individual. Such an approach would move beyond traditional phenotype classification and integrate multiple dimensions of patient care, including comorbidity burden, cardiovascular status, inflammatory activity, vascular access characteristics, body composition, residual kidney function, treatment tolerance, symptom burden, and patient-reported outcomes [30,31,32].

From this perspective, convective volume itself may represent only one component of a personalized treatment strategy. Achieving high convection volumes remains an important therapeutic objective, but the capacity to deliver and sustain these volumes varies substantially among patients. Treatment time, blood-flow rate, vascular access performance, ultrafiltration requirements, sodium and volume management, and intradialytic hemodynamic stability all influence the effective delivery of OL-HDF and may modify its clinical impact. Personalization may therefore depend less on selecting patients for OL-HDF than on tailoring treatment prescriptions to maximize the benefits of convective therapy while minimizing treatment burden and adverse effects. This view is consistent with recent proposals advocating precision dialysis rather than uniform dialysis delivery models [31,32].

Advances in data science and individualized treatment-effect modelling may further support this transition toward precision dialysis. Rather than relying on conventional subgroup analyses, future studies may combine clinical variables, laboratory biomarkers, treatment parameters, and longitudinal outcomes to identify patient-specific patterns of benefit. Artificial intelligence and machine-learning approaches may provide additional opportunities to develop adaptive dialysis strategies tailored to the needs of individual patients [31,33].

Equally important is the need to expand the outcomes used to evaluate treatment effectiveness. Most studies examining OL-HDF have focused primarily on mortality, yet many of the proposed phenotypes discussed in this review are characterized by concerns extending beyond survival alone. Functional status, symptom burden, hemodynamic tolerance, recovery time after dialysis, quality of life, hospitalization, and maintenance of independence may be particularly relevant outcomes for older, frail, and cardiovascularly vulnerable patients. A precision-medicine approach should therefore incorporate patient-centred outcomes alongside conventional clinical endpoints.

Ultimately, the search for a single “ideal” OL-HDF phenotype may be less informative than the development of individualized strategies that integrate patient characteristics, treatment goals, and technical feasibility. The future of OL-HDF may therefore lie not in identifying who should receive convective therapy, but in determining how to deliver the right convective therapy to the right patient at the right time. Such a shift from phenotype-based selection toward precision dialysis may represent the next stage in the evolution of OL-HDF.

Despite the accumulating evidence supporting high-volume OL-HDF, several clinically relevant questions remain unanswered. Future studies should move beyond traditional subgroup analyses and investigate whether combinations of clinical characteristics, biological markers, and treatment-related variables can identify patients who derive the greatest benefit from specific OL-HDF prescriptions.

One of the main research priorities is the identification of mechanisms underlying the observed clinical benefits associated with high-volume OL-HDF. A better understanding of the relative contribution of middle-molecule removal, inflammation modulation, hemodynamic stability, volume management, and cardiovascular protection may help refine treatment strategies and guide patient-centred treatment goals.

Future investigations should also focus on the incorporation of biomarkers into dialysis research. Inflammatory markers, indicators of biological ageing, cardiovascular biomarkers, and measures of uraemic toxin burden may provide complementary information beyond conventional clinical variables and contribute to a more comprehensive characterization of treatment response.

Advances in artificial intelligence and machine-learning methodologies may further accelerate the transition toward precision dialysis. The integration of clinical, biological, and treatment-related data may facilitate the development of predictive models capable of supporting individualized treatment decisions and optimizing OL-HDF prescriptions in routine clinical practice.

In addition, greater emphasis should be placed on outcomes that matter most to patients. While mortality remains a key endpoint, future studies should systematically evaluate quality of life, symptom burden, post-dialysis recovery time, physical function, cognitive performance, frailty progression, and maintenance of independence. These outcomes may be particularly relevant in older and medically complex dialysis populations.

Finally, prospective studies are needed to determine how treatment parameters such as convection volume, treatment time, vascular access performance, and fluid-management strategies can be optimized according to individual patient needs. Such research may ultimately help optimize treatment delivery and maximize the benefits of convective therapies across diverse clinical settings.

## 12. Conclusions

Current evidence does not support restricting OL-HDF to any specific patient phenotype. Randomized evidence supports an overall clinical benefit of OL-HDF in the populations studied, while phenotype-specific differences in treatment effect remain largely unproven. Clinical characteristics may nevertheless influence baseline risk, potential absolute benefit, treatment tolerability, technical feasibility, and the ability to achieve effective convective therapy.

In that sense, although high-volume OL-HDF shows consistent benefits in the populations included in randomized trials, these findings apply primarily to patients who meet trial eligibility criteria and in whom adequate convection volumes can be achieved safely and effectively. Therefore, treatment decisions should be individualized according to clinical characteristics, treatment goals, vascular access performance, technical feasibility, and the ability to deliver effective convective therapy.

Ultimately, precision medicine in OL-HDF may depend less on identifying which patients should receive convective therapy than on individualizing how convective therapy is prescribed and delivered according to each patient’s clinical characteristics, treatment goals, and technical limitations.

## Figures and Tables

**Table 1:** Potential clinical phenotypes and practical considerations for personalization of high-volume OL-HDF.

Clinical Scenario	Rationale for OL-HDF	Key Prescription Priorities	Current Evidence and Remaining Uncertainties	Evidence Level
**Older patients [6,7,8,9,10,11]**	High baseline cardiovascular risk and potential absolute clinical benefit	Prioritize treatment tolerance, with adequate convective volume and individualized goals rather than maximal convection alone	No evidence of a treatment-by-age interaction; greater absolute benefit may result from higher baseline risk	Indirect evidence from randomized studies
**Anuric patients [12]**	Complete dependence on extracorporeal clearance and greater inflammatory burden	Maximize effective convective clearance whenever technically feasible	Biological rationale exists; no evidence of a specific treatment interaction	Indirect clinical/observational evidence
**Patients with heart failure and cardiovascular vulnerability [7,8,10]**	Volume overload, myocardial stress, and cardiovascular risk	Integrate convection with individualized sodium prescription, careful ultrafiltration and hemodynamic stability	Strong biological rationale; dedicated randomized evidence is lacking	Indirect clinical/observational evidence
**Patients with poor hemodynamic tolerance [13,14]**	Improved intradialytic stability and symptom burden	Combine OL-HDF with cooling strategies, individualized ultrafiltration and careful volume management	Benefit probably reflects a multimodal strategy rather than OL-HDF alone	Indirect clinical/observational evidence
**Frailty and biological vulnerability [15,16,17,18]**	Reduced physiological reserve and high susceptibility to dialysis stress	Emphasize tolerance, recovery after dialysis and patient-centred outcomes	Clinical rationale exists; direct evidence remains limited	Hypothesis-generating
**Patients with inflammation and high uremic burden [19,20,21]**	Enhanced removal of middle molecules and inflammatory mediators	Deliver adequate convection while monitoring nutritional status and albumin losses when appropriate	Improvements demonstrated mainly for surrogate biomarkers	Hypothesis-generating
**Incident dialysis patients [22,23,24,25,26,27]**	Opportunity for early exposure to convective therapy before cumulative dialysis injury develops	Consider early implementation of high-volume OL-HDF whenever technically feasible	Evidence derived from observational studies; randomized confirmation needed	Hypothesis-generating

Abbreviations: OL-HDF, online hemodiafiltration.

**Table 2:** Evidence for treatment-effect modification across patient subgroups in randomized studies of online hemodiafiltration.

Subgroup	HDF IPD Meta-Analysis [1]	ESHOL [8]	CONVINCE [9]	Interpretation
**Age**	Interaction *p* = 0.09	Interaction *p* = 0.129	Assessed; no significant interaction reported	Possible trend toward effect modification, not consistently demonstrated
**Sex**	Interaction *p* = 0.72	Interaction *p* = 0.792	Assessed; no significant interaction reported	No evidence of effect modification
**Diabetes**	Interaction *p* = 0.95	Interaction *p* = 0.757	Subgroup analyses reported; interaction test not reported	No consistent evidence of effect modification
**Cardiovascular disease**	Interaction *p* = 0.82	NR	Subgroup analyses reported; interaction test not reported	No consistent evidence of effect modification
**Albumin**	Interaction *p* = 0.85	NR	NR	No evidence of effect modification
**Vascular access**	Interaction *p* = 0.16	Interaction *p* = 0.776	Assessed; no significant interaction reported	No evidence of effect modification
**Dialysis vintage**	Interaction *p* = 0.58	NR	Assessed; no significant interaction reported	No evidence of effect modification
**Residual kidney function**	NR	NR	Assessed; no significant interaction reported	No evidence of effect modification
**Comorbidity burden**	No equivalent subgroup available	Interaction *p* = 0.022	NR	Isolated signal, not replicated

Values shown are interaction *p* values from published subgroup analyses when available. NR = not reported. Subgroup-specific effect estimates and 95% confidence intervals are provided in Table 2 when reported in the original publications.

## Data Availability

No new data were created or analyzed in this study. Data sharing is not applicable to this article.
